# Acoustic, Mechanical and Thermal Properties of Green Composites Reinforced with Natural Fibers Waste

**DOI:** 10.3390/polym12030654

**Published:** 2020-03-13

**Authors:** Tufail Hassan, Hafsa Jamshaid, Rajesh Mishra, Muhammad Qamar Khan, Michal Petru, Jan Novak, Rostislav Choteborsky, Monika Hromasova

**Affiliations:** 1 Faculty of Textile Engineering, National Textile University, Faisalabad 37610, Pakistan; tufailhassan12@gmail.com (T.H.); qamarkhan154@gmail.com (M.Q.K.); 2 Protective Textile Group, National Textile University, Faisalabad 37610, Pakistan; 3 Faculty of Engineering, Czech University of Life Sciences Prague, Kamýcká 129, 165 00 Praha-Suchdol, Czech Republic; choteborsky@tf.czu.cz (R.C.); hromasova@tf.czu.cz (M.H.); 4 Department of Machinery Construction, Institute for Nanomaterials, Advanced Technology and Innovation, Technical University of Liberec, Studentska 2, 46117 Liberec, Czech Republic; michal.petru@tul.cz; 5 Faculty of Mechanical Engineering, Technical University of Liberec, Studentska 2, 46117 Liberec, Czech Republic; jan.novak1@tul.cz

**Keywords:** sound absorption coefficient, impact strength, flexural strength, coefficient of thermal expansion, bio-composite

## Abstract

The use of acoustic panels is one of the most important methods for sound insulation in buildings. Moreover, it has become increasingly important to use green/natural origin materials in this area to reduce environmental impact. This study focuses on the investigation of acoustic, mechanical and thermal properties of natural fiber waste reinforced green epoxy composites. Three different types of fiber wastes were used, e.g., cotton, coconut and sugarcane with epoxy as the resin. Different fiber volume fractions, i.e., 10%, 15% and 20% for each fiber were used with a composite thickness of 3 mm. The sound absorption coefficient, impact strength, flexural strength, thermal conductivity, diffusivity, coefficient of thermal expansion and thermogravimetric properties of all samples were investigated. It has been found that by increasing the fiber content, the sound absorption coefficient also increases. The coconut fiber-based composites show a higher sound absorption coefficient than in the other fiber-reinforced composites. The impact and flexural strength of the cotton fiber-reinforced composite samples are higher than in other samples. The coefficient of thermal expansion of the cotton fiber-based composite is also higher than the other composites. Thermogravimetric analysis revealed that all the natural fiber-reinforced composites can sustain till 300 °C with a minor weight loss. The natural fiber-based composites can be used in building interiors, automotive body parts and household furniture. Such composite development is an ecofriendly approach to the acoustic world.

## 1. Introduction

The word noise is derived from the Latin word “nausea” which means the sensation of discomfort. Noise is the unwanted sound, it may be loud, distracting or annoying. Noise pollution refers to the unwanted sound waves in the environment produced by humans that becomes a threat to the health of both human and animals. Noise has become the third largest source of pollution which causes diverse environmental effects on the economy and human health [1]. Advancement in technology and lifestyle has, in many ways, caused an increase in air, soil, water and noise pollution [2]. Sound absorption takes place when sound waves strike the surface of any porous material. Some of the sound is reflected back while some waves or energy are absorbed by the material [3]. Vehicular transportation is one of the major sources for producing noise pollution. Various types of materials are used to prevent noise pollution like wall cladding, acoustic barriers and acoustic ceilings which cover a wide range of applications [4]. Verma et al. described that composites, ceramics and plastics are vastly used for acoustic panels as compared to other materials. Traditionally, carbon and glass fiber-reinforced composites were used for acoustic absorbance. The advantage of synthetic fiber-reinforced composite is its high strength and mechanical properties which are more suitable to use in structural applications. The main problems associated with synthetic fiber-reinforced composites are the environmental aspects, e.g., production process, application and afterlife disposal. It is harmful to the environment because it is not biodegradable and made from nonrenewable resources [5].

The most important thing is to protect our environment from pollution, and it can be achieved without compromising the performance and quality of the product. The solution is to use biodegradable materials which are obtained from natural and renewable sources. Due to environmental concern, plant fiber-reinforced composites are receiving greater attention of researchers and industrialists because they are biodegradable, combustible and lightweight [6]. 

Traditionally, noise is controlled by non-biodegradable and expensive materials such as polymer fibers, glass wool, fabric fillers and polymer foams. Mineral fibers like mineral wool and glass fibers are widely used for the manufacturing of soundproofing materials in buildings and industries but both of them are harmful to the environment and risky to human health [7]. In the 1970s, public health regulations banned the use of carcinogenic materials, which enabled the transition of soundproofing materials from asbestos to synthetic fibers. These fibers are non-biodegradable, cause heavy pollution and have a high carbon footprint which is harmful to the environment. Along with synthetic fibers, wood is the most desirable candidate for industries to manufacture sound-absorbing materials with relatively good physical and mechanical properties. Due to its diverse characteristics, wood has a huge demand for various industries, which causes shortages in the supply of wood. A large number of trees are being cut annually for the manufacturing of acoustic panels causing unrecoverable damage to our ecosystem. In such scenarios, different industries, especially acoustic material industries, must begin to search for a suitable and environmentally friendly substitute that can fulfill the demand. Currently, the most commonly used materials for sound absorption are fiber-reinforced polymer composites, e.g., glass fibers composites. In addition, polystyrene, poly(vinyl chloride), polyurethane and poly(acrylic ester)s are some polymers that are widely used by the acoustic industries. These materials are either expensive or hazardous during normal use [8,9,10]. Sound absorption performance can be divided into several classes based on the sound absorption coefficients as shown in Table 1. 

Recently, natural fiber-reinforced composites have received great attention from researchers and industrialists as a replacement of synthetic fiber-reinforced composite. They have relatively good mechanical and physical characteristics that can be used in various applications. Natural fibers are bio-degradable, nonabrasive, nonhazardous, lightweight and renewable materials. The most commonly used natural fibers as a reinforcement in composites are hemp, flax, luffa, banana, jute, sisal, sugarcane, ramie, betel nut, etc. [12]. Various studies show that natural fibers, e.g., tea leaf, rice-straw, coconut and kenaf fibers have a very good capability of sound absorption and are widely used in industries for manufacturing of sound-absorbing panels [13,14,15,16,17].

A lot of work has been done on natural fiber-reinforced composites so far by different researchers. Chen et al. investigated the sound absorption coefficient of ramie fiber-reinforced polylactic acid composites. They found that the sound absorption coefficient is ranged between 0.089 and 0.353 in a frequency range of 250–1600 Hz [18]. Wang established in his research that the sound absorption properties of rock wool are found to be similar to that of glass fibers [19]. 

Khusairy et al. investigated the acoustic properties of banana fiber-reinforced epoxy composites in the frequency range of 500–6000 Hz. The sound absorption coefficient was found to be 0.11. They further described that by changing fiber diameter, flow resistivity changes hence it causes a significant change in sound absorption coefficient [20]. Chen et al. described that the sound absorption coefficient of ramie fiber-reinforced polylactic acid is found to be 0.121 in the frequency range between 500 and 6000 Hz [18]. Bratu et al. used different waste materials, e.g., steelworks slag, fiberglass waste, wood waste, waste ash from the burning shells of oil seeds from plants as reinforcement in formaldehyde composite. They found excellent results of sound absorption coefficient (SAC) 0.8–0.9 in the frequency range of 400–3200 Hz [21]. Yang et al. investigated ramie, flax and jute reinforced epoxy composites. SAC of these fibers is found to be between 0.6 and 0.65 at a frequency range of 2000 Hz. They further described that multiscale surface structure and hollow lumen of natural fiber is mainly responsible for high SAC [1]. Abdullah et al. investigated sugarcane bagasse fiber and banana fiber-reinforced polyester resin composites. They found that the banana fiber composite has SAC 0.6835 while bagasse fiber has SAC 0.6338 at 4000 Hz frequency. They also prepared hybrid composites from these fibers and found that SAC is greater than the individual fiber component [22]. Jayamani et al. investigated the acoustic properties of rice-straw/polypropylene and kenaf/urea formaldehyde composites. They found that the SAC of rice-straw/polypropylene composite is 0.008 and kenaf/urea composite is 0.065, respectively, at 2000 Hz frequency [23]. 

Moretti et al. in 2016 investigated the acoustic and thermal properties of mineral fiber basalt in order to use it in the application of building and construction. The sound reverberation method was used for checking the acoustic properties. They found that with increasing frequency of incident sound waves, sound absorption properties also tend to improve. Further, they found that basalt fibers exhibit relatively good sound absorption and thermal insulation properties, hence they are best-suited materials for panels in building and construction [24]. 

In 2015, Jayamani et al. investigated the SAC of sisal fiber-reinforced polylactic acid composites. At a frequency of 2000 Hz, they show SAC of 0.085. They further found that by increasing fiber content, SAC also increases [25]. The same researchers, in another work, investigated the acoustic behavior of different natural fiber-reinforced epoxy composites. They used coconut/coir, sugarcane bagasse and kenaf fibers as reinforcement and found that the SAC of the coconut/coir reinforced composite is 0.078, sugarcane bagasse reinforcement is 0.075 and kenaf reinforcement 0.078, respectively, at 6000 Hz frequency. Coconut/coir reinforced composite shows higher SAC than the sugarcane bagasse composite because coconut fiber is more porous as compared to sugarcane bagasse which causes increased SAC [26].

Ricciardi et al. designed the acoustic panels manufactured from recycled materials like paper waste and textile fiber waste as reinforcement and bonded them with glue, making samples of 2–20 mm thickness. They measured SAC in frequency ranges of 100–6400 Hz through the transfer function impedance tube. They found that by increasing the thickness of samples, SAC also increases [27]. 

Jiang et al. investigated the seven-hole hollow polyester fibers (SHPF) reinforced chlorinated polyethylene (CPE) composites. It has been observed that for 3 mm thickness of a sample, SAC was 0.695 at frequency 2500 Hz. Further by increasing the fiber content SAC also increases [28]. Zhang et al. investigated different fibers, e.g., flax, carbon, glass, ramie and jute for reinforcement of epoxy (Bisphenol-A) composites. These fibers are used in the form of plain-woven fabric in the manufacturing of composites. It was found that the flax, ramie and jute reinforced composites have higher SAC than carbon and glass reinforced composite for a wider frequency range up to 10,000 Hz [29]. Zhang et al. investigated the acoustic properties of flax and basala wood reinforced composites at 250–10,000 Hz range. They found that the SAC of the of flax reinforced composites is 0.96 and that of the basala wood reinforced composite is 0.58 [30]. 

The use of wood and synthetic fiber/polymers composites for sound insulation applications is not environmentally friendly. Abundant cutting of trees is done for wood, whereas synthetic fibers/polymer composites have a non-degradability issue. Therefore, there is an urgent need to work in this area and develop relatively economic, biodegradable and environmentally friendly sound absorption material. It will greatly help the saving of trees and will reduce the use of non-biodegradable materials for acoustic applications. The overall impact will be to protect our environment from several pollution hazards including noise.

## 2. Materials and Methods

### 2.1. Materials

**Figure 1:**
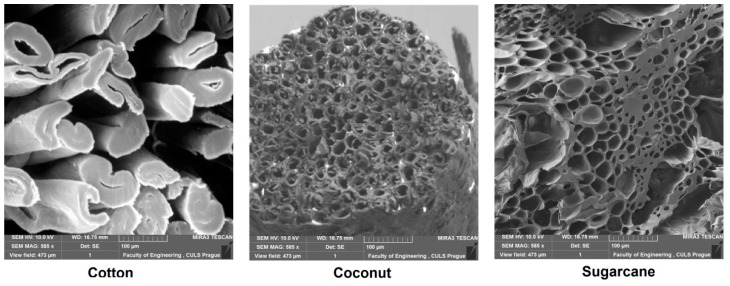
Microscopic images of fiber cross-sections.

Three different types of fiber wastes, e.g., cotton fly, coconut/coir husk and sugarcane waste were used in the manufacturing of composite samples. Cotton fly was collected from a yarn manufacturing industry, coconut husk was purchased from a local market and sugarcane fibers were extracted from bagasse. The cross-sectional images of the fibers were taken by microscopy and shown in Figure 1. 

The properties and chemical composition of these fibers are given in Table 2 and Table 3, respectively.

Green epoxy resin CHS-G530 (new commercial name EnviPOXY^®^530) was purchased from company SPOLCHEMIE in Ústí nad Labem, Czech Republic. It has low molecular weight and contains low crude oil content and high renewable content. Chemically it contains 4,4′-Isopropylidenediphenol, oligomeric reaction products with 1-chloro-2-3-epoxypropane. It is called “green” due to obtaining 1-chloro-2-3-epoxypropane (epichlorohydrine) from glycerine originating from waste generated at biodiesel production. Its properties are given in Table 4.

### 2.2. Methods

#### 2.2.1. Fiber Preparation

In the case of sugarcane, the waste of sugarcane bagasse was collected from a juice extractor. Joints were removed and treatment of the bagasse was done by adding 4 g/L sodium hydroxide in water. The bagasse was immersed in NaOH solution for three hours. The fibers were separated from treated sugarcane manually and cut into a short length of approximately 3–7 mm. Coconut fibers were opened and then cut into short lengths ranging from 3 to 9 mm. Cotton fly having a length of 12–15 mm fiber was used for the manufacturing of composite.

#### 2.2.2. Samples Preparation

**Figure 2:**
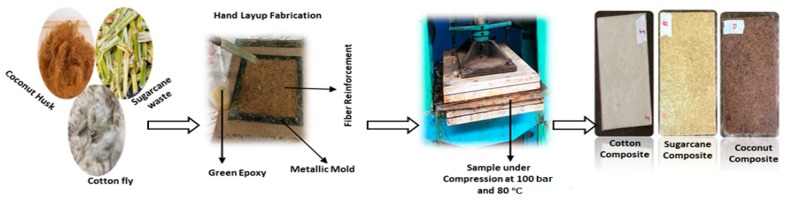
Illustration scheme for fabrication of composite samples.

In order to develop composite samples, a mold of 20 cm × 20 cm (length × width) and 3 mm thickness was prepared. Fibers were uniformly distributed within the boundaries of the mold. In order to ensure uniform fiber distribution, the spreading was done layer by layer so that the averaging effect will minimize the variation of mass. This method was especially beneficial at low fiber content. No visible voids were found in the prepared samples. A mixture of epoxy and hardener (CHS-HARDENER P11) was prepared as per manufacturer (SPOLCHEMIE) guidelines with a ratio of 100:32 and stirred well for uniform mixing. The mixture was dispensed over the fibers in the mold very carefully to ensure uniform distribution of epoxy throughout the samples. A Teflon sheet was placed on both sides of the material in order to keep them in hydraulic press machine model TDF-110 under 100 bar pressure for 30 min at 80 °C. It was followed by curing of the sample at room temperature. In total, nine types of samples were developed by repeating the same procedure with fiber content 10%, 15% and 20% for all three types of fibers. The samples were cut in the size for different characterization as per standard requirement (Figure 2).

### 2.3. Characterization

#### 2.3.1. Acoustic Testing

In order to measure the sound absorption coefficient (SAC), two microphone impedance tube (Brüel & Kjær, model 4206, Nærum, Denmark) with a diameter of 100 mm was used in a frequency range 0 Hz to 1600 Hz as per ASTM E1050-08 standard. The samples were cut with a 140 mm diameter. Ten measurements were carried out and the average was reported. Graphs were plotted for SAC with respect to fiber content and fiber type. 

#### 2.3.2. Impact Test

To investigate the impact properties of the samples, Charpy impact tester from Zwick/Roell HIT 50P, Ulm, Germany were used according to ISO-179-1 standard. Samples were cut into size 80 mm × 10 mm for testing. Ten measurements were carried out and the average was reported.

The thickness and width of the samples were measured by Vernier caliper before the test. Specimens were placed on the specific slot and the pendulum was allowed to impact with 50 J energy in order to hit and break the specimen. The absorbed energy was recorded, and impact strength was calculated using Equation (1)

(1) ∂cu = WB/bh ×103,

where WB, is the energy at break, in joules; b, is the width of the specimens in millimeters; h, is thickness of the specimens in millimeters.

#### 2.3.3. Flexural Strength Testing

In order to investigate the flexural strength of composites, the samples were cut into size 13 mm × 120 mm and the test was performed by universal testing machine Zwick/Roell Z100 by ASTM D 7264 standard. The gauge length of 80 mm, extension rate of 1 mm/min and load of 5 kN was used. The flexural strength was calculated by the Equation (2)

(2) $$\sigma \;=3\mathrm{PL}/2{\mathrm{bh}}^{2},$$

where P represents load; L represents gauge length; b represent width; h represents thickness. Ten measurements were carried out and the average was reported.

#### 2.3.4. Light Microscopy

The light microscope MIC-209 was used to capture images of impact broken samples with 20% fiber content from all three types of composites. The images were taken to check the mode of fiber failure, i.e., slippage or rupture of fibers in composites during impact testing.

#### 2.3.5. Thermal Properties

C-Therm thermal conductivity analyzer (TCi) was used to measure the thermal properties like conductivity, resistance and diffusivity of the samples. The C-Therm (Tci) thermal conductivity analyzer allows determining accurate values for thermal conductivity and thermal effusivity of materials without extensive sample preparation or damage to the sample.

This highly accurate technique is based on the transient plane source (TPS) method. The primary difference between the traditional and modified transient plane source techniques is that the modified method offers a single-side interface compared to the double-sided interface requirements of the traditional version. 

The modified transient plane source (MTPS) technique has many advantages in comparison to other available testing methods, e.g., guarded hot plate, hot wire or hot probe. The non-invasive nature of the C-Therm TCi’s MTPS sensors allows testing of materials of any size in-situ or in laboratories without destruction of the specimen. Moreover, testing can be done in seconds with consistent and accurate results. 

The TCi consists of a sensor, power control device and computer software. A spiral-type heating source is located at the center of the sensor where heat is generated. The generated heat enters the material through the sensor during which a voltage drop occurs rapidly at the heating source. The thermal conductivity is calculated through the voltage drop data. Before conducting the measurements, all samples were conditioned at standard atmospheric conditions (25 ± 2 °C, 65% ± 2% RH) for 24 h. The average of ten measurements for each sample was taken and the mean values of the thermal properties were calculated. The tested data were statistically analyzed.

#### 2.3.6. Linear Coefficient of Thermal Expansion

The increase or decrease in the length of any material by changing temperature is represented by the linear coefficient of thermal expansion (LCTE). Measurement of LCTE is very important in order to know the dimensional changes of materials at different temperatures and one can decide the specific end-use of a material in terms of temperature condition. Dilatometer DIL 2010STD (Orton, Westerville, OH, USA) was used to measure the LCTE of specimens with high sensitivity using the ASTM E831 standard. The setup consists of three main components:

- Sample holder and push rod;
- Furnace or Chamber;
- Linearly Variable Differential Transformer (LVDT) for measuring changes in dimensions.

The sample holder and push rod support the thermocouple and the sample. Different varieties of the sample holder are available like silica or alumina etc. Furnace provides heat to the specimens in vacuum. LVDT is used as a measuring head which measures the dimensional changes of the specimen with respect to temperature. Samples were cut into size 2.54 cm × 1 cm. A temperature range of 40–120 °C was used during the test with a 5 °C/min increasing rate.

#### 2.3.7. Thermogravimetric Analysis

To investigate the thermal stability of the samples, NETZSCH TG 209F1 Libra instrument TGA having crucible weight 139 g (NETZSCH-Gerätebau GmbH, Selb, Germany) and sample weight 2 g was used. Weight loss with increasing temperature was measured in a temperature range of 25–300 °C with an increasing rate of 10 °C/min.

## 3. Results and Discussion

### 3.1. Acoustic Properties

#### 3.1.1. Effect of Fiber Type on Sound Absorption Coefficient (SAC)

The effect of different types of fiber reinforcement in composites on the sound absorption coefficient was studied. The main aim of the current research is to develop green composite materials from natural fiber waste which is impregnated with green epoxy resin. The targeted products are to be used in lightweight construction, indoor panels and automotive components. Thus, the mechanical, thermal and stability at higher temperature are essential properties which are investigated.

In all the above applications, there is an essential requirement of sound absorption. Therefore, a composite panel with some acoustic performance is an added advantage. It may be pointed out that the prepared composite panels have some sound insulation as compared to the pure green epoxy panel which shows absolutely no absorption at all. The increase of SAC with the increase of fiber volume fraction is demonstrated.

**Figure 3:**
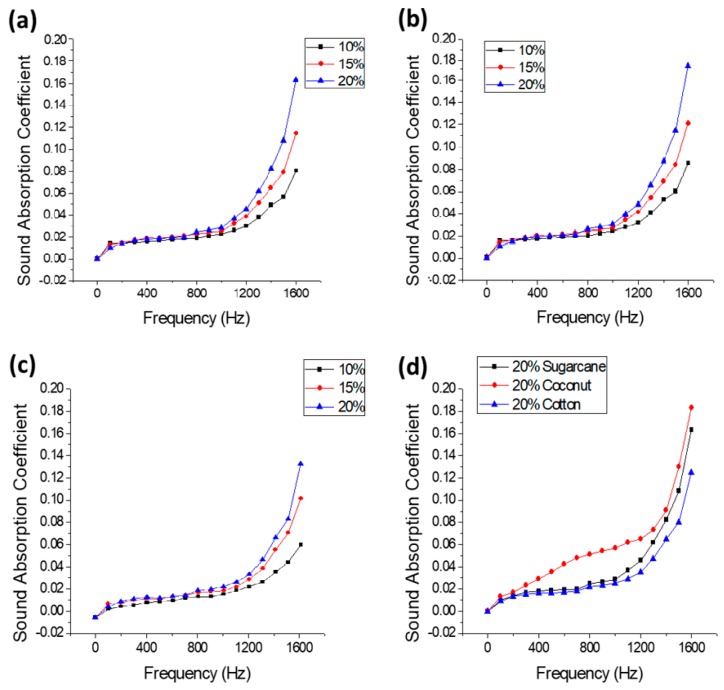
(**a**) Effect of fiber content on the sound absorption coefficient (SAC) of the sugarcane fiber-based composites. (**b**) Effect of fiber content on SAC of the coconut fiber-based composites. (**c**) Effect of fiber content on SAC of the cotton fiber-based composites. (**d**) Effect of fiber type on acoustic properties of composites with 20% fiber content.

The results revealed that the coconut fiber-reinforced composites show the highest SAC followed by sugarcane fiber-reinforced composites and cotton fiber-reinforced composites, respectively, as shown in Table 5 and Figure 3d.

The sugarcane fiber-reinforced composites have a higher SAC than the cotton fiber-reinforced composites; this is because the SAC of the fibers mainly depends upon their porosity. Large hollow lumen structure of fibers has the ability to absorb sound more efficiently than small lumen structures. It must be noted that the overall hollow lumen of the coconut fiber is larger than of sugarcane and cotton. Sugarcane fibers have a larger cumulative lumen diameter than cotton lumen diameter. Previously, researchers investigated and established that larger lumen structure has the ability to absorb more sound than small lumen structure. Overall, the SAC of materials under the present study is relatively lower and therefore, they can be used as good sound blockers rather than absorbers. 

#### 3.1.2. Effect of Fiber Content on Sound Absorption Coefficient

The effect of fiber content on the sound absorption coefficient was studied in the frequency range of 0–1600 Hz. A systematic increase in SAC is observed with an increase of fiber content as shown in Table 5 and Figure 3a–c. It is due to the fact that higher fiber volume fraction allows the sound waves to pass through a more tortuous path causing higher friction, thus higher energy loss takes place. Furthermore, natural fibers have a porous surface and internal structure with a hollow lumen which helps in the dissipation of sound energy [31]. 

### 3.2. Mechanical Properties

#### 3.2.1. Effect of Fiber Type on Impact Strength of Composites

**Figure 4:**
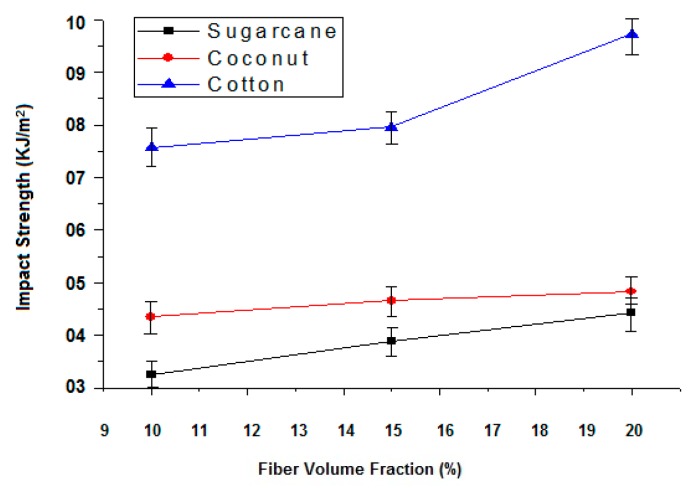
Effect of fiber type and fiber content on the impact properties of the sugarcane, coconut and cotton fiber-reinforced composites.

The ability of the material to resist fracture when load is applied at high speed is known as impact strength. In composites with epoxy resin, the curing process enables the formation of a complex 3D network which increases the mechanical performance substantially. The impact properties of the composites with cotton, coconut and sugarcane fibers have been investigated. There is a substantial improvement of impact strength as compared to that of pure green epoxy. The results revealed that the cotton fiber-reinforced composites have relatively higher impact strength than the sugarcane and coconut fiber-reinforced composites as shown in Table 6 and Figure 4.

It should be noted that tenacity of the cotton fiber is higher than the sugarcane and coconut fiber as shown in Table 2. Previously researchers found that high tenacity and long fiber length leads to an increase in impact strength [32]. Further, the coconut fiber-reinforced composites have higher impact strength than the sugarcane fiber-based composites because the tenacity of the coconut fiber is greater than that of the sugarcane fiber. 

#### 3.2.2. Effect of Fiber Content on Impact Strength of Composites

**Figure 5:**
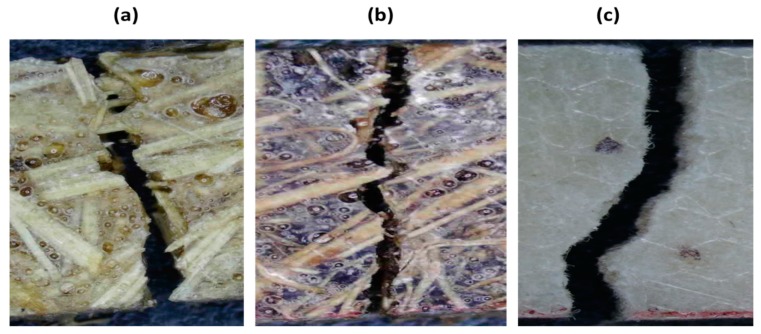
Microscopic images of impact broken samples: (**a**) sugarcane (**b**) coconut (**c**) cotton fiber-reinforced composites.

Results revealed that when increasing fiber content, impact strength also increases (Figure 5). It has been observed that 10%, 15% and 20% of the sugarcane fiber content shows an impact strength of 3.25 kJ/m^2^, 3.89 kJ/m^2^ and 4.43 kJ/m^2^, respectively. Overall, a 36% increase in impact strength is observed with the increase of fiber content from 10% to 20%. Similarly, an 11% increase in impact strength is observed, when the coconut fiber content is increased from 10% to 20%. In the case of the cotton fiber-reinforced composites, there is a 28% increase in impact strength when the fiber content increases from 10% to 20%. In a fiber-reinforced composite system, the reinforcement is the main constituent which is responsible to bear the impact load. Similar observations have been made by several other researchers [33,34]. 

#### 3.2.3. Effect of Fiber Type on Flexural Strength of Composites

**Figure 6:**
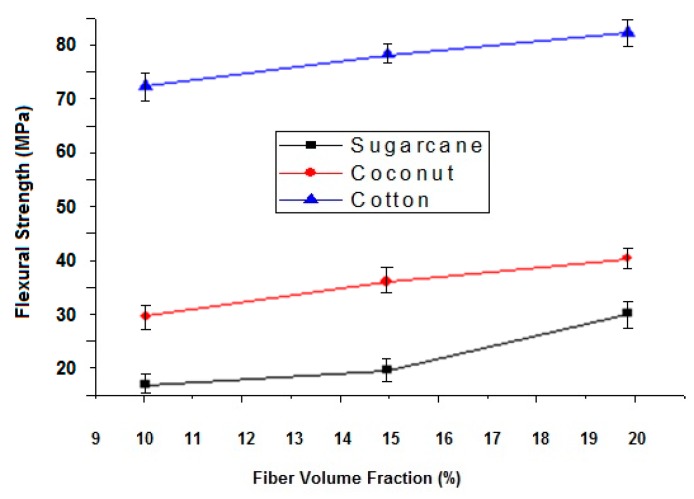
Effect of fiber type and fiber content on flexural strength of the sugarcane, coconut and cotton fiber-reinforced composites.

The ability of a material to resist bending deformation under load is called flexural strength. It mainly depends upon the type of reinforcement and matrix. The results of the study revealed that the flexural strength of the cotton fiber-reinforced composite is higher than the sugarcane fiber and coconut fiber composites as shown in Table 7 and Figure 6. It can be justified based on the fact that the tenacity of the cotton fibers is higher than the coconut and sugarcane fibers. It is also well known that bending rigidity is directly proportional to the tensile modulus. Researchers have previously reported that by increasing the tenacity of the fiber, the flexural strength of the composites can be increased [35,36]. 

#### 3.2.4. Effect of Fiber Content on Flexural Strength of Composites

It has been observed that when increasing fiber content, flexural strength also increases. The flexural rigidity has a direct proportionality with the modulus of elasticity or tenacity. The fiber reinforcement is the major load-bearing element in a composite system. By increasing the fiber volume fraction (FVF) from 10% to 20%, there is a substantial increase in flexural strength. FVF can be further increased by impregnation using the infusion method. As the set up used in the current investigation was a relatively simpler one, there was a restriction to maximum FVF and preparing void-free samples. Previously, researchers have found a significant increase in flexural strength by increasing fiber content in composites [37,38].

### 3.3. Thermal Properties

#### 3.3.1. Effect of Fiber Type and Fiber Content on Thermal Conductivity, Resistance and Diffusivity

The thermal properties evaluated by the C-Therm thermal conductivity analyzer (TCi) are given in Table 8.

##### Thermal Conductivity

Thermal conductivity, λ, is a measure of the rate at which heat is transferred through the unit area of the sample across unit thickness under a specified temperature gradient and thus is defined by the relation below.

(3) $$\lambda \left[Wm^{-1}K^{-1}\right]=\frac{Q}{Ft\frac{\Delta T}{h}},$$

$\lambda \left({\mathrm{Wm}}^{-1}K^{-1}\right)=\frac{Q}{F\tau \frac{\Delta T}{h}}$ where Q is the amount of conducted heat, F is the area through which heat is conducted, *t* is the time of heat conduction, ∆T is the drop of temperature and h is the sample thickness. 

**Figure 7:**
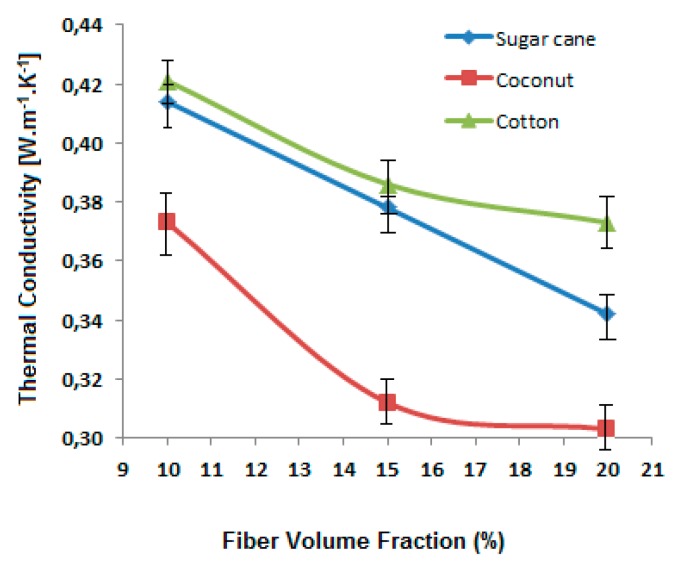
Effect of fiber type and fiber content on thermal conductivity of the sugarcane, coconut and cotton fiber-reinforced composites.

The thermal conductivity is a material intrinsic property and dependent on its porosity (volume of air content). The higher the density, the higher the conductivity. The pure epoxy resin shows a higher conductivity as compared with the fiber-reinforced composites. It is due to the fact that all fibers have a significant volume of air entrapped inside their structure. The comparison of the thermal conductivity of the reinforced composites is shown in Figure 7.

It is observed that thermal conductivity decreases with increasing content of the fiber reinforcement. It is because of the fact that the fibers have lower conductivity themselves in comparison with the matrix (green epoxy resin and hardener). Among the three types of fibers, cotton fiber-reinforced composites show higher conductivity due to higher fiber density and relatively lower porosity of the cotton fibers as compared to coconut and sugarcane. Further, the porosity in coconut fibers is much higher due to the overall lumen area which is responsible for the lowest thermal conductivity.

##### Thermal Diffusivity

**Figure 8:**
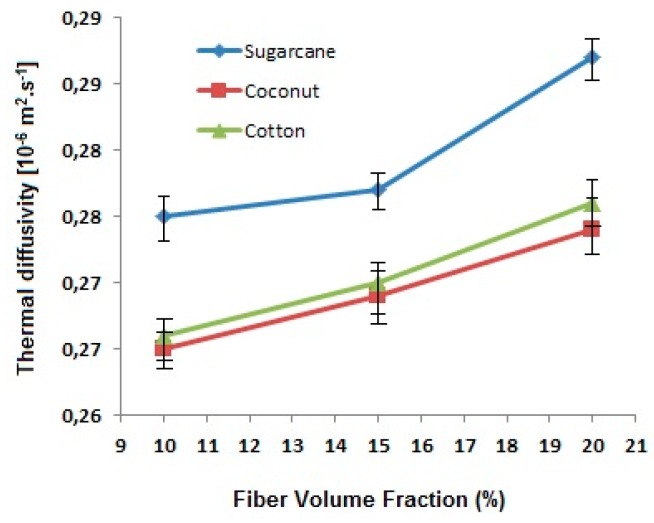
Effect of fiber type and fiber content on thermal diffusivity of the sugarcane, coconut and cotton fiber-reinforced composites.

Thermal diffusivity describes the rate of heat spread through a material. The fiber-reinforced composite panels show improved diffusivity in comparison to pure green epoxy resin. The comparison of thermal diffusivity for the reinforced composites is shown in Figure 8.

It is found that the composites improve diffusivity due to the fibrous reinforcement. A higher volume fraction of fibers leads to an increase in thermal diffusivity. Among the fiber types investigated, the sugarcane-based composite panels diffuse heat more significantly in comparison with coconut and cotton-based composites. This may be attributed to the chemical composition of sugarcane which is rich in hemicellulose and lignin.

##### Thermal Resistance

Thermal resistance is defined as the ratio of the temperature difference between the two faces of a material to the rate of heat flow per unit area. Thermal resistance determines the heat insulation property of a material. The higher the thermal resistance, the lower is the heat loss. The thermal resistance, R, is connected with the thermal conductivity, λ, and the thickness, h, as follows.

(4) $$R\left[m^{2}K^{-1}W^{-1}\right]=\frac{h}{\lambda }.$$

**Figure 9:**
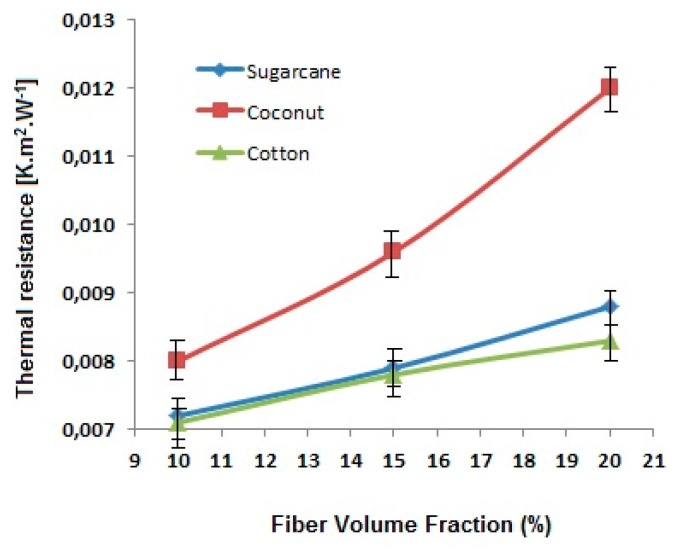
Effect of fiber type and fiber content on thermal resistance of the sugarcane, coconut and cotton fiber-reinforced composites.

Thus, thermal resistance is critically dependent on thickness and thermal conductivity. As all the composite panels have almost the same thickness, the resistance is inversely proportional to the conductivity. The fiber-reinforced composites have higher thermal resistance as compared to pure epoxy resin. The comparison of thermal resistance for the reinforced composite panels is shown in Figure 9.

It is observed that thermal resistance increases linearly when increasing the fraction of the reinforcing fiber. Among the three types of fibers used, coconut fiber has a relatively higher porosity and thus the lowest thermal conductivity. This leads to the highest thermal resistance among the fiber types investigated.

#### 3.3.2. Effect of Fiber type on Coefficient of Thermal Expansion in Composites

**Figure 10:**
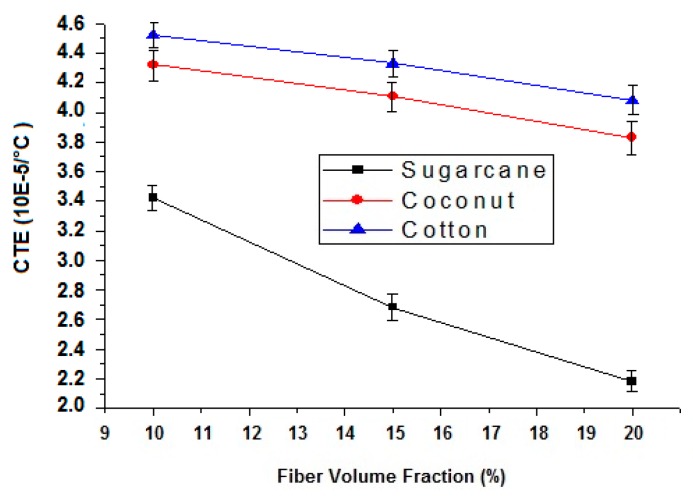
Effect of fiber type and content on thermal expansion coefficient of composites.

Thermal expansion is basically the property of a material to change its length, area, volume or shape due to an increase in temperature. By increasing temperature, the average kinetic energy of the molecules increases, and the molecular vibration also increases. The relative expansion of material divided by the temperature is known as the coefficient of thermal expansion. It has been observed that the thermal expansion coefficient of the cotton fiber-reinforced composite is higher than both the sugarcane and coconut fiber composite as shown in Table 9 and Figure 10. The coconut fiber composite has a higher coefficient of thermal expansion (CTE) than the sugarcane fiber composite. It might be based on the chemical composition of the constituent fibers. The percentage of cellulose content in cotton is higher than the coconut and sugarcane fibers as shown in Table 3. Cellulose inherently has a linear structure and could thermally expand to a greater extent than hemicellulose and lignin. The structure of lignin contains aromatic rings which increase thermal stability. Researchers have investigated the thermal degradation of cellulose, hemicellulose and lignin of sugarcane bagasse. It is found that the thermal stability of lignin is higher than of cellulose and hemicellulose. Furthermore, the thermal stability of hemicellulose is higher than of cellulose [39]. 

#### 3.3.3. Effect of Fiber Content on Coefficient of Thermal Expansion of Composites

It has been observed that, in all cases, increasing the fiber content decreases the coefficient of thermal expansion as shown in Table 9 and Figure 10. It is due to the fact that the thermal expansion of epoxy (matrix) is much higher than fibers (reinforcement) therefore by increasing fiber content (FVF), results in decreasing in CTE [40,41]. By lowering the coefficient of thermal expansion, the composite materials will deform to a relatively smaller extent in high-temperature applications.

#### 3.3.4. Thermogravimetric Analysis (TGA)

**Figure 11:**
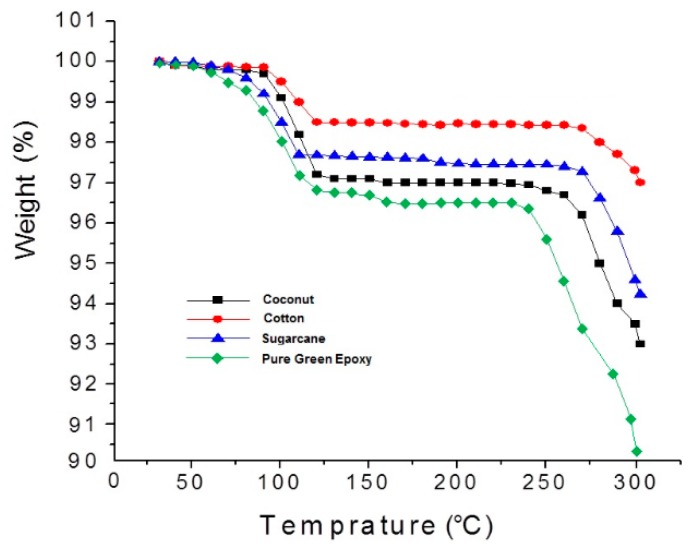
TGA analysis of the cotton, coconut and sugarcane reinforced epoxy composite with a 15% fiber volume fraction (FVF).

The weight loss of composite as a function of increasing temperature was measured by TGA. Untreated fiber contains hemicellulose and lignin thus it can store water. It has been observed that weight loss starts from 80 to 115 °C which indicates dehydration of fibers as shown in Figure 11. The results revealed that the cotton, coconut and sugarcane fiber-reinforced composites show initial degradation and weight loss of 1.5%, 2.8% and 2.3%, respectively. There is no significant weight loss in the samples at a temperature range of 115 to 270 °C. Significant weight loss is observed at 270–300 °C, which corresponds to the degradation of the matrix. Furthermore, the selected reinforcement fibers contain cellulose and it starts degradation around 270 °C [35]. The coconut, sugarcane and cotton fiber-reinforced composites show a relatively smaller overall weight loss of 7%, 5.8% and 3%, respectively, at 300 °C. It must be pointed out that the fiber-reinforced composites show lower weight loss as compared to the pure green epoxy which shows a 9.8% weight loss. The thermal stability of the fiber-reinforced composites is sensitive to the weight content of reinforcing fibers, rather than their volume content. At the same volume fraction, the weight content is dependent on the density of constituent fibers. As the cotton fiber has a higher density as compared to coconut and sugarcane, the weight fraction is substantially higher and the effect is clearly reflected in TGA results. The weight loss is inversely proportional to the cellulose content in these fiber types. The non-cellulosic constituents degrade at relatively lower temperatures resulting in higher weight loss in coconut and sugarcane fiber-reinforced composites. However, cotton fiber-reinforced composites can survive for a relatively higher temperature range due to a high content of cellulose which is thermally more stable [40,41].

## 4. Conclusions

The effect of different cellulosic fibrous waste and their content (FVF) on acoustic, mechanical and thermal properties of green/bio epoxy composites has been investigated. The results reveal that an increase in fiber content tends to increase the sound absorption coefficient. Compared to pure epoxy resin, the SAC of the fiber-reinforced composites is higher. As most of the natural cellulosic fibers are highly porous in nature, they have much higher SAC compared to the matrix phase, therefore SAC increases with increasing fiber content. Among the samples investigated, coconut/coir fiber-reinforced composites show the highest sound absorption coefficient followed by sugarcane and cotton fiber-reinforced composites for 10%, 15% and 20% fiber content based on overall porosity of the constituent fibers. Fiber porosity has a direct relation with sound absorption. 

Impact strength, as well as flexural strength, increases by increasing fiber content in all the cellulosic fiber types because the fiber/reinforcement phase is the main load-bearing constituent in a composite system. Among the samples investigated, cotton fiber-reinforced composites have the highest impact and flexural strength followed by coconut and sugarcane fiber composites. It is due to higher tenacity of the cotton fiber as compared to both coconut fiber and sugarcane fiber. 

An increase in fiber content decreases the thermal conductivity and thus increases thermal resistance. It is because of the fact that the fibers have lower conductivity themselves in comparison with the matrix (green epoxy resin and hardener). Among the three types of fibers, cotton fiber-reinforced composites show higher conductivity due to higher fiber density and relatively lower porosity of the cotton fibers as compared to coconut and sugarcane. Further, the porosity in coconut fibers is much higher due to the overall lumen area which is responsible for the lowest thermal conductivity. It is found that the composites improve thermal diffusivity due to the fibrous reinforcement. A higher volume fraction of fibers leads to an increase in thermal diffusivity. Among the fiber types investigated, the sugarcane-based composite panels diffuse heat more significantly in comparison with coconut and cotton-based composites. This may be attributed to the chemical composition of sugarcane which is rich in hemicellulose and lignin.

The coefficient of thermal expansion (CTE) decreases with an increase in fiber content because the CTE of epoxy resin is much higher than the reinforcing cellulosic fibers. Furthermore, cotton fiber-reinforced composites show higher CTE compared to coconut and sugarcane fibers due to high cellulose content. Cellulose is thermally more sensitive than hemicellulose and lignin. Thermogravimetric analysis reveals that the composite with 15% of the coconut, sugarcane and cotton fiber content show 7%, 5.8% and 3% weight loss, respectively, at 300 °C, while pure green epoxy shows a weight loss of 9.8%. 

Based on the performance analysis of the samples in the current study, coconut fiber-reinforced composites are the most suitable materials among all the three types investigated as far as acoustic performance is concerned. On the other hand, higher tenacity of the cotton fiber enables it to be the best reinforcement when mechanical performance, e.g., impact strength and flexural strength are desired. Further, the higher cellulose content in the case of cotton proves to be thermally more stable (minimum weight loss) as compared to the coconut and sugarcane fiber-based counterparts, especially at elevated temperatures. However, cotton tends to expand more in the linear direction. The composites will change dimension but can survive hot conditions. The possible applications of proposed composites are in building construction, indoor panels, and automotive body parts with some sound insulation. For more effective and efficient sound insulation, the composite panels themselves are not sufficient and additional porous fibrous layers can be added on top of composite panels. They can be used as a separator and as a possible replacement of pure/virgin wood in household furniture, etc. 

## Figures and Tables

**Table 1:** Classes of sound absorption coefficient [11].

Sound Absorption Classes	Sound Absorption Coefficient Range
A	0.90–1.00
B	0.80–0.85
C	0.60–0.75
D	0.30–0.55
E	0.15–0.25
F	0.00–0.10

**Table 2:** Fiber properties.

Fiber Type	Fiber Fineness (Tex)	SD (±)	Fiber Length (mm)	Force at Break (cN)	SD (±)	Elongation at Rupture (mm)	SD (±)	Tenacity (cN/Tex)	SD (±)
Sugarcane	36	1.12	3–7	330.6	14.22	1.32	0.04	9.2	0.27
Coconut	32	0.83	3–9	360.6	14.43	4.52	0.13	11.2	0.56
Cotton Fly	0.19	0.007	12–15	2.70	0.11	3.5	0.09	14.2	0.66

SD: Standard Deviation.

**Table 3:** Chemical composition of fibers.

Fiber Type	Cellulose (%)	Hemicellulose (%)	Lignin (%)	Pectin (%)	Wax (%)
Sugarcane	28.3–50	20–36.3	21.2–24	N/A	0.9
Coconut	19.9–55	11.9–15.4	32.7–53.3	4.7–7	N/A
Cotton	82.7	5.7	28.2	5.7	0.6

**Table 4:** Properties of green epoxy resins.

Properties	Value
Physical State	Liquid at 20 °C
Color	Yellowish to Yellow
Boiling Point	270–280 °C (at very low pressure)
Density (g/cm^3^)	1.16 at 20 °C
Water Solubility (g/L)	6–9 at 20 °C
Viscosity (Poise)	8–10 at 25 °C
Solubility	Soluble in Acetone
Storage Temperature	5–25 °C
Epoxide Index (mol/kg)	5.4–5.7
Mass Equivalent of Epoxide, EEW (g/mol)	176–186
Color (Hz, G*)	Max. 100
Hydrolyzable Chlorine Content (%)	Max. 0.03
Non-Volatile Substances (2 h/140 °C)	Min. 99.5%

**Table 5:** Sound absorption coefficient of samples at 1600 Hz frequency.

Reinforcement Type	Fiber Volume Fraction (%)	Fiber Weight Fraction (%)	SAC at 1600 (Hz)	SD (±)
**Pure Green Epoxy**	0	0	0.000	0.000
**Sugarcane**	10	4.75	0.081	0.002
	15	7.13	0.115	0.004
	20	9.50	0.163	0.005
**Coconut**	10	6.12	0.090	0.002
	15	9.18	0.130	0.005
	20	12.24	0.183	0.006
**Cotton**	10	14.25	0.059	0.001
	15	21.38	0.097	0.004
	20	28.50	0.125	0.006

SD: Standard Deviation.

**Table 6:** Impact strength of samples.

Reinforcement Type	Fiber Volume Fraction (%)	Fiber Weight Fraction (%)	Impact Strength (KJ/m^2^)	SD (±)
**Pure Green Epoxy**	0	0	2.75	0.12
**Sugarcane**	10	4.75	3.25	0.15
	15	7.13	3.89	0.17
	20	9.50	4.43	0.18
**Coconut**	10	6.12	4.35	0.18
	15	9.18	4.66	0.17
	20	12.24	4.83	0.22
**Cotton**	10	14.25	7.57	0.28
	15	21.38	7.96	0.37
	20	28.50	9.73	0.38

SD: Standard Deviation.

**Table 7:** Flexural properties of samples.

Reinforcement Type	Fiber Volume Fraction (%)	Fiber Weight Fraction (%)	Flexural Strength (MPa)	SD (±)
**Pure Green Epoxy**	0	0	15.4	0.76
**Sugarcane**	10	4.75	16.6	0.77
	15	7.13	19.3	0.79
	20	9.50	29.7	1.03
**Coconut**	10	6.12	29.2	1.12
	15	9.18	35.6	1.34
	20	12.24	39.9	1.50
**Cotton**	10	14.25	71.9	2.98
	15	21.38	77.6	3.25
	20	28.50	81.7	4.03

SD: Standard Deviation.

**Table 8:** Thermal properties of samples.

Reinforcement Type	Fiber Volume Fraction (%)	Fiber Weight Fraction (%)	Thermal Conductivity (W·m^−1^·K^−1^)	SD (±)	Thermal Diffusivity (10^−6^ m^2^·s^−1^)	SD (±)	Thermal Resistance (K·m^2^·W^−1^)	SD (±)
**Pure Green Epoxy**	0	0	0.435	0.02	0.262	0.012	0.0069	0.00021
**Sugarcane**	10	4.75	0.414	0.03	0.275	0.012	0.0072	0.00026
	15	7.13	0.378	0.01	0.277	0.013	0.0079	0.00033
	20	9.50	0.342	0.02	0.287	0.015	0.0088	0.00037
**Coconut**	10	6.12	0.373	0.01	0.265	0.011	0.0080	0.00032
	15	9.18	0.312	0.01	0.269	0.012	0.0096	0.00048
	20	12.24	0.303	0.01	0.274	0.012	0.0120	0.00007
**Cotton**	10	14.25	0.421	0.02	0.266	0.013	0.0071	0.00029
	15	21.38	0.386	0.02	0.270	0.014	0.0078	0.00042
	20	28.50	0.373	0.01	0.276	0.013	0.0083	0.00038

SD: Standard Deviation.

**Table 9:** Coefficient of thermal expansion of developed samples.

Reinforcement Type	Fiber Volume Fraction (%)	Fiber Weight Fraction (%)	CTE (10^−5^/°C)	SD (±)
**Pure Green Epoxy**	0	0	6.342	0.32
**Sugarcane**	10	4.75	3.422	0.16
	15	7.13	2.681	0.14
	20	9.50	2.182	0.12
**Coconut**	10	6.12	4.323	0.24
	15	9.18	4.114	0.16
	20	12.24	3.832	0.18
**Cotton**	10	14.25	4.519	0.28
	15	21.38	4.333	0.28
	20	28.50	4.084	0.18

SD: Standard Deviation.
